# Insight Into Rho Kinase Isoforms in Obesity and Energy Homeostasis

**DOI:** 10.3389/fendo.2022.886534

**Published:** 2022-06-13

**Authors:** Lei Wei, Jianjian Shi

**Affiliations:** Herman B Wells Center for Pediatric Research, Department of Pediatrics, Indiana University, School of Medicine, Indianapolis, IN, United States

**Keywords:** ROCK, beige adipogenesis, energy expenditure, obesity, ROCK isoform-selective

## Abstract

Obesity and associated complications increasingly jeopardize global health and contribute to the rapidly rising prevalence of type 2 diabetes mellitus and obesity-related diseases. Developing novel methods for the prevention and treatment of excess body adipose tissue expansion can make a significant contribution to public health. Rho kinase is a Rho-associated coiled-coil-containing protein kinase (Rho kinase or ROCK). The ROCK family including ROCK1 and ROCK2 has recently emerged as a potential therapeutic target for the treatment of metabolic disorders. Up-regulated ROCK activity has been involved in the pathogenesis of all aspects of metabolic syndrome including obesity, insulin resistance, dyslipidemia and hypertension. The RhoA/ROCK-mediated actin cytoskeleton dynamics have been implicated in both white and beige adipogenesis. Studies using ROCK pan-inhibitors in animal models of obesity, diabetes, and associated complications have demonstrated beneficial outcomes. Studies *via* genetically modified animal models further established isoform-specific roles of ROCK in the pathogenesis of metabolic disorders including obesity. However, most reported studies have been focused on ROCK1 activity during the past decade. Due to the progress in developing ROCK2-selective inhibitors in recent years, a growing body of evidence indicates more attention should be devoted towards understanding ROCK2 isoform function in metabolism. Hence, studying individual ROCK isoforms to reveal their specific roles and principal mechanisms in white and beige adipogenesis, insulin sensitivity, energy balancing regulation, and obesity development will facilitate significant breakthroughs for systemic treatment with isoform-selective inhibitors. In this review, we give an overview of ROCK functions in the pathogenesis of obesity and insulin resistance with a particular focus on the current understanding of ROCK isoform signaling in white and beige adipogenesis, obesity and thermogenesis in adipose tissue and other major metabolic organs involved in energy homeostasis regulation.

## Introduction

Obesity is known as a major risk factor for type 2 diabetes, cardiovascular disease and several cancers, with obvious and significant impacts on public health. In 2016, over 1.9 billion adults and 340 million minors worldwide aged 5-19 were overweight or obese (1–7). In 2020, 39 million children under the age of 5 were overweight or obese. As the result of excess body mass, 2.8 million people die each year, and an additional estimated 35.8 million (2.3%) of global disability-adjusted life years (DALYs) are affected (7). More recently, during the COVID-19 pandemic, there has been a sharp increase in BMI among young school-aged children aged 2–19 years described in a recent longitudinal cohort study (8). These findings highlight the importance of integrating obesity management effort into public health interventions in present day. On the other hand, we must clearly appreciate that obesity is a preventable global health issue; therefore developing novel therapies to stop excessive adipose tissue expansion can significantly contribute to public health.

White adipose tissue (WAT) not only stores energy in the form of fat, but also displays important endocrine function in metabolic homeostasis (9). In contrast, brown adipose tissue (BAT) and beige adipocytes are thermogenic and can convert glucose and fatty acids into heat, thereby acting as thermogenic tissue to increase energy expenditure (10). BAT is mainly found in the dorsal interscapulum region in rodents and characterized by high constitutive expression of thermogenic genes, most notably: uncoupling protein-1 (UCP1), which resides in the inner mitochondrial membrane of brown adipocytes (11). UCP1 mediates heat generation in brown adipocytes by uncoupling respiratory chain, thus causing a fast substrate oxidation without efficient ATP production. In addition, clusters of “brown-like” UCP1-positive cells, also known as beige adipocytes, develop in WAT in response to various activators including cold exposure and β-adrenergic stimulation. BAT is also found in adult humans, but it is mainly characterized as inducible beige fat described in rodents (12, 13). Various factors may apply negative effects on brown adipocytes regeneration leading to its decreased prevalence as humans age (14). It is worth noting that increased thermogenic activities in either brown or beige adipocytes have been linked to obesity resistance which were reported in numerous studies involving mouse models (9, 15–17) and humans (18–24). Increasing energy expenditure through the formation and activation of brown and beige adipocytes is an electrifying pathway with great potential in reducing obesity.

One strategy is to target molecules involved in the adipocyte formation and differentiation process. Rho kinases (hereafter referred to as ROCKs) are major downstream effectors of the small GTPase RhoA (25–28). The ROCK family has recently emerged as a potential therapeutic target for metabolic disorders. In human and mouse, both ROCK1 and ROCK2 are ubiquitously expressed across tissues (27). ROCKs play central roles in the organization of the actin cytoskeleton and is involved in a wide range of fundamental cellular functions such as smooth muscle cell contraction, cell proliferation, adhesion, migration, polarity, cytokinesis, differentiation and survival in many cell types. ROCK activity change has been demonstrated in pathogenesis of various diseases. Up-regulated ROCK activity is implicated in the pathogenesis of all aspects of metabolic syndrome including obesity, insulin resistance, dyslipidemia and hypertension (29–33). RhoA/ROCK-mediated cytoskeleton changes are identified as important regulators in adipogenesis (29, 34–37) and insulin sensitivity (30, 38–42). Studies using ROCK inhibitors in animal models of obesity, diabetes, and associated complications have demonstrated beneficial outcomes (29, 43–53). However, most ROCK inhibitors in these studies are non-isoform selective, thereby limiting their therapeutic potential due to observed dose-dependent smooth muscle relaxation and vascular hypotension in systemic treatment, which obviously hampers ROCK inhibition as a novel treatment (54–56). Thus, development of isoform-selective inhibitors can help speed up a new strategy breakthrough.

In addition to previous metabolic studies utilizing non-selective, exogenously administered chemical inhibitors of ROCK, studies which utilize genetically modified animal models to identify isoform selective roles of ROCK in the pathogenesis of metabolic disorders including obesity, have also been of particular interest to medical scientific community. Although, earlier published studies in the past decade were focused on ROCK1 activity (33, 57–59), a growing interest in understanding ROCK2 isoform function has emerged more recently with the development of ROCK2-selective inhibitors (54–56). For example, KD025 (also known as SLx-2119), a selective ROCK2 inhibitor, shows no major side effects in various clinical trials focusing on non-metabolic diseases (60–63). Hence, studying individual ROCK isoforms to elucidate their unique roles and mechanisms in white and beige adipogenesis, insulin sensitivity, and energy balance will facilitate significant breakthroughs in systemic application of isoform-selective inhibitors for obesity prevention and treatment. In this review, we give an overview of ROCK functions in the pathogenesis of obesity with a particular focus on the current understanding of ROCK isoform signaling involved in white and beige adipogenesis, obesity development and thermogenesis in adipose tissue and other major metabolic organs involved in energy homeostasis regulation.

## Overview of Major ROCK Signaling Pathways in Regulating Metabolic Function

### Regulation of ROCK Activity in Metabolic Tissues

ROCK1 and ROCK2 are downstream targets of the small GTP-binding protein RhoA, they work as mediators in the RhoA-dependent signaling pathway. Stimulation of tyrosine kinase and G-protein-coupled receptors leads to activation of RhoA *via* the recruitment and activation of guanine nucleotide exchange factors (GEFs) (64, 65). Activated RhoA directly interacts with ROCK at the C-terminal portion of the coiled-coil domain and induces a conformational change, leading to activation of the serine/threonine kinase toward selective substrates (25–28). ROCK activity can also be modulated through interaction of C-terminal pleckstrin-homology domain with lipid mediators and with the plasma membrane (66–68), auto-phosphorylation through dimerization (69), and proteolytic cleavage of the inhibitory C-terminal domain (70–72). In addition, recent microRNA (miRNA) research has identified numerous miRNAs which are involved in regulating RhoA and ROCK expression and kinase activity (73, 74), suggesting that miRNAs have a very important position in RhoA/ROCKs gene regulation.

**Figure 1:**
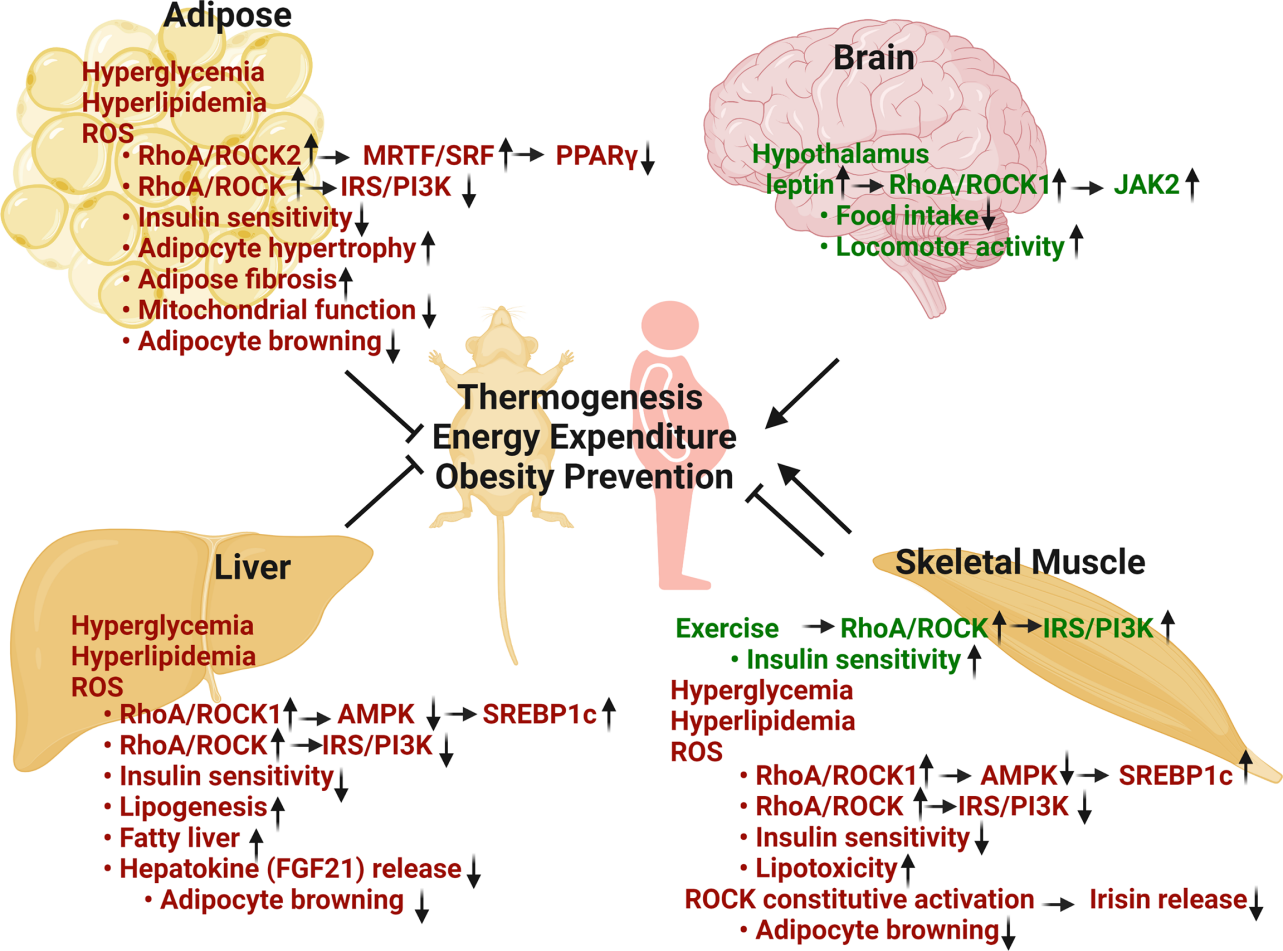
Metabolic regulation of RhoA/ROCK pathway in thermogenesis, energy balance, obesity prevention and insulin sensitivity. Adipose RhoA/ROCK activation, caused by metabolic stresses including hyperglycemia, hyperlipidemia and ROS, is associated with disease phenotypes. Total ROCK activity and ROCK2 activity are negative regulators of thermogenic, adipogenic, and mitochondrial gene expression *via* stimulation of F-actin/MRTF/SRF signaling pathway and inhibition of insulin signaling pathway. Hepatic RhoA/ROCK activation by risk-factors is also associated with aberrant regulation. Total ROCK activity and ROCK1 activity are positive regulators of lipogenic pathway through inhibition of AMPK and activation of SREBP1c. Hepatic RhoA/ROCK activities are also negative regulators of thermogenesis and insulin signaling pathway. Skeletal muscle RhoA/ROCK activation shows both beneficial and detrimental outcomes. ROCK activation under basal or exercise condition is critical to glucose regulation *via* promoting insulin signaling pathway. Conversely, ROCK activation by metabolic stresses is associated with disease through inhibition of AMPK and insulin signaling. Hypothalamic RhoA/ROCK1 activation by circulating leptin decreases body weight and adiposity through reducing food intake, increasing energy expenditure and locomotor activity (Green texts indicate beneficial effects in metabolism; red texts indicate detrimental effects in metabolism) (Figure is created with BioRender.com).

Both experimental (29, 43–45) and clinical studies (31, 41, 75) have established a clear link between aberrant ROCK activity and metabolic disease including obesity and insulin resistance (**Figure 1**). The factors that can change kinase activity and/or gene expression are context dependent, as indicated in hyperglycemia, hyperlipidemia, mechanical stress, oxidative stress and increased production of inflammatory cytokines etc. (29, 31, 41, 43–45, 75) (**Figure 1**). Metabolic stresses including hyperglycemia and hyperlipidemia mainly stimulate RhoA/ROCK activity *via* oxidative stress-dependent pathways and through activation of GEFs by tyrosine kinase and G-protein-coupled receptors; moreover, other protein kinases including adenosine monophosphate-activated protein kinase (AMPK) can activate RhoA (29–33, 76). Recent mechanistic studies which comprehensively link hyperglycemia and hyperlipidemia with aberrant RhoA/ROCK activity have shown that the increased production of proinflammatory cytokines and oxidative stress can disturb miRNA expression which subsequently change RhoA/ROCK expression and activity as well (77, 78). miRNAs were found in metabolic tissues including adipose tissue (79), liver (80, 81), skeletal muscle (82) and kidney (83), et al., where they regulate RhoA, ROCK1 and ROCK2 expression and activity (**Table 1**). Although miRNAs are known as a class of regulatory molecules, elucidating their biological relevance of individual miRNAs is challenging. Their up- or down-regulations can be either protective or detrimental to insulin resistance (82), diabetes (83) and obesity (79–81). Despite their elusive role, miRNAs modify RhoA/ROCK signaling pathway and obviously contribute to animal development, metabolic homeostasis, and disease pathogenesis.

**Table 1:** Down**-**regulation of RhoA, ROCK1 and ROCK2 by miRNAs in metabolic tissues.

Regulated Transcript	miRNA	Function	Pathology	Ref
RhoA	miR-124	Neurogenesis of adipose derived of mesenchymal stromal cells	Cell therapy for neurological diseases	(84)
	miR-125a-3p	Adipogenesis of subcutaneous WAT	Multiple symmetric lipomatosis	(79)
	miR-133a	Contractility of gastric smooth muscle	Diabetic abnormal gastric emptying	(78)
	miR-133b	Proliferation and apoptosis of human retinal endothelial cells	Diabetic retinopathy	(85)
	miRNA-141	Proliferation and apoptosis of the penile cavernous smooth muscle cells	Diabetic erectile dysfunction	(86)
	miR-142-5p	Neurogenesis and proliferation of adipose derived of mesenchymal stromal cells	Cell therapy for neurological diseases	(87)
ROCK1	miR-135a	Insulin signaling in skeletal muscle cells	Insulin resistance	(88)
	miR-145	Proliferation and migration of VSMCs	Diabetic atherosclerosis	(89)
	miR-146a-5p	Lipid accumulation in liver	Nonalcoholic fatty liver disease	(80)
	miR-148b	Insulin signaling in skeletal muscle cells	Insulin resistance	(82)
	miR-202	Insulin signaling in skeletal muscle cells	Insulin resistance	(88)
	miR-214	Insulin signaling in skeletal muscle cells	Insulin resistance	(88)
	miR-217	Proliferation and migration of VSMCs	Diabetic atherosclerosis	(90)
	miR-324-5p	Glucose and lipid metabolism in liver	Diabetes and obesity	(81)
ROCK2	miR-10a	Contractility of VSMCs	Diabetes and hyperlipidemia	(77)
	miR-139b	Contractility of VSMCs	Diabetes and hyperlipidemia	(77)
	miR-455-3p	Glomerular hypertrophy, mesangial proliferation, and renal fibrosis	Diabetic nephropathy	(83)
ROCK1&2	miR-497	Endothelial-to-mesenchymal transition of glomerular endothelial cells	Diabetic nephropathy	(91)

### ROCKs Regulate Actin Cytoskeleton Function in all Metabolic Tissues

**Figure 2:**
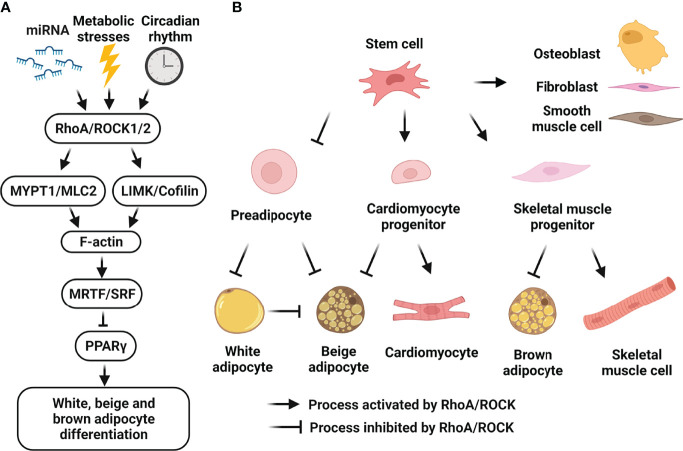
Cellular roles of RhoA/ROCK signaling in white, beige and brown adipocyte differentiation. **(A)** Schematic representation of RhoA/ROCK pathway which negatively regulates adipocyte differentiation. RhoA/ROCK signaling-cascade activation can be induced by many risk-factors including mechanical stress, mitochondrial dysfunction, inflammation, ROS, aging, hyperglycemia and dyslipidemia, etc. Activated pathway causes actin polymerization and formation of stress fibers, leading to nuclear translocation of MRTF and suppression of PPARγ activity. **(B)** Schematic representation of the regulation of cell fate determination of mesenchymal stem cells by RhoA/ROCK pathway. Activation of RhoA/ROCK signaling by local mechanical stresses and environment factors promotes the commitment to osteogenic *versus* adipogenic lineages of bone marrow mesenchymal stem cells and adipose tissue-derived stem cells. RhoA/ROCK activation also promotes the commitment to smooth muscle cell-like and fibrogenic lineages *versus* adipogenic lineage of perivascular progenitors. Moreover, RhoA/ROCK activation favors a switch toward myogenesis *versus* brown adipogenesis of common mesenchymal precursors, and a switch toward cardiomyocytes *versus* beige adipocytes of embryonic cardiac progenitors. Finally, RhoA/ROCK activation negatively regulates preadipocyte differentiation towards white and beige adipocytes, and transdifferentiation of white adipocytes to beige adipocytes. (Figure is created with BioRender.com).

ROCKs are downstream of the small GTPase RhoA and regulate actin cytoskeleton function as central regulators (25–27). The two ROCK isoforms are highly homologous with 92% amino acid sequence identity in the kinase domain and an overall shared identity of 65% (25–27). ROCK1 and ROCK2 share more than 30 immediate downstream substrates (reviewed in refs (73, 92–94)) which share the consensus amino acid sequences: R/K-X-S/T or R/K-X-X-S/T (R, arginine; K, lysine; S, serine; T, threonine, all potential phosphorylation sites by ROCK) (95, 96). The major downstream substrates of ROCKs include the myosin binding subunit of myosin light chain (MLC) phosphatase-1 (MYPT1) (95, 97, 98), MLC2 (97, 99) and LIM kinases (LIMK) (96, 100–103). ROCKs/MYPT1/MLC2 pathway promotes actomyosin contractility through MLC2 phosphorylation, and ROCKs/LIMK/cofilin pathway stabilizes actin filaments (F-actin) through cofilin phosphorylation, which inhibits actin-depolymerization activity (**Figure 2A**). These two signaling pathways modulate actin cytoskeleton organization, stress fiber formation and smooth muscle cell contraction, and remain the best characterized mechanism presenting in all metabolic tissues.

### MRTF and SRF are Downstream Mediators Mainly in Adipose and Vascular Tissues

The prominent effects of RhoA/ROCK on cytoskeleton dynamics are not only limited to cell contraction, adhesion, morphology and motility, but also include transcriptional regulation. For instance, serum response factor (SRF) activity is regulated by RhoA/ROCK signaling on actin polymerization (104–107). Myocardin, the myocardin-related transcription factors A and B (MRTF-A/MKL1 and MRTF-B/MKL2) and MASTR constitute an SRF coactivator family, and their activity depends on actin dynamics (106–109). Association of MRTF-A with monomeric globular actin (G-actin) results in its sequestration in the cytoplasm, and actin polymerization leads to MRTF-A translocation into the nucleus and SRF target gene activation (106, 107). The RhoA/ROCK/F-actin/MRTF-A/SRF pathway has been well-established in smooth muscle cells and regulates transcription of genes involved in the contractile function of smooth muscle cells (110).

Recent studies also demonstrate the role of this pathway in regulating lipid and glucose metabolisms (111, 112). In adipose tissue, MRTF-A/SRF pathway suppresses peroxisome proliferator-activated receptor γ (PPARγ) expression/activity and negatively regulates adipocyte differentiation including white (37, 113), beige (16, 114) and brown adipogenesis (115) (**Figure 2**). Furthermore, activation of this pathway was found to reduce expression of insulin receptor substrate-1 (IRS1) and PPARγ in mature human adipocytes and in hypertrophic adipocytes from ob/ob mice, thus contributing to adipocyte dysfunction and insulin resistance (116). This pathway also promotes fibrosis, inflammation and insulin resistance in diet-induced obesity, since perivascular progenitor cells will preferentially differentiate into a fibroblast lineage as opposed to a normal adipocyte lineage (117). Even in mature adipocytes, this pathway promotes their fibroblast-like phenotype contributing to adipocyte dysfunction and mitochondrial dysfunction (118). In addition to adipose tissue, MRTF-A/SRF pathway is involved in hepatic fibrosis (119) and diabetic nephropathy (120): MRTF-A knockout reduced macrophage infiltration, and ameliorated inflammation and fibrosis of liver tissues in mice with nonalcoholic steatohepatitis induced by high-fat diet (HFD) (119); through an epigenetic mechanism, MRTF-A deficiency also attenuated connective tissue growth factor in diabetic nephropathy (120). It should be noted some metabolic studies cited above were interested in the connection between RhoA/ROCK/F-actin and MRTF-A/SRF pathways (16, 37, 114, 116), but some mainly focused on MRTF-A and its downstream effectors (113, 115, 117). Together, besides the well-established role for the RhoA/ROCK/F-actin/MRTF-A/SRF pathway in regulating vascular cell function, abovementioned studies support that the RhoA/ROCK/F-actin/MRTF-A/SRF pathway negatively regulates adipocyte differentiation, plays a negative role in maintaining adipose tissue health, and contributes to liver fibrosis and diabetic nephropathy.

### Impacts of ROCKs on Insulin Signaling

The relationship between ROCK activity and IRS1 phosphorylation has been documented in various cell types and organs including adipose tissues, liver, skeletal muscle and vascular system (**Figure 1**). It is widely believed that serine phosphorylation of IRS1 by ROCK activity in WAT and vascular cells leads to reduced IRS1-mediated phosphatidylinositol 3-kinase (PI3K) activation, resulting in decreased insulin sensitivity (30, 34, 42, 43, 121, 122). However, ROCK-mediated IRS1 phosphorylation can also positively impact on insulin signaling such as in skeletal muscle, and in cultured adipocytes and muscle cells (38–41). Although it has been consistently reported that ROCK activation increases serine phosphorylation levels of IRS1 (Ser307 and Ser632/635) and IRS2 (39, 122–124), the effects on insulin-stimulated tyrosine phosphorylation of IRS1 and IRS2, and on PI3K activation appear to be complex and context-dependent (38). It is also noted that ROCK-mediated actin cytoskeleton dynamics and insulin signaling can be inter-regulated; for instance, the changes of actin dynamics in ROCK1 deficient MEFs could be linked to improved insulin signaling through increased insulin receptor (IR) activation (30). Therefore, these studies support the concept that ROCK activity has both positive and negative actions on insulin signaling in a cell-type and tissue-dependent manner.

### AMPK and SREBP are Downstream Mediators Mainly in Liver and Skeletal Muscle

AMPK is a key regulator of glucose and fatty acid metabolism and plays a major role in obesity and type 2 diabetes. The relationship between ROCK and AMPK has been documented in various cell types and organs, although physical interaction of these molecules has not been identified (125). ROCK1 has been identified as an upstream negative regulator of AMPK activity through an unknown mechanism in liver (47, 48, 59, 80, 125), skeletal muscle (47, 48, 126) and pancreatic β-cells (127). In liver tissue, ROCK1/AMPK/sterol regulatory element-binding protein-1c (SREBP1c) axis was reported to regulate hepatic lipogenesis and contribute to nonalcoholic fatty liver diseases (59, 125) (**Figure 1**). In skeletal muscle cells, ROCK1/AMPK/SREBP1c axis was involved in lipotoxicity and insulin resistance (126) (**Figure 1**). In pancreatic β-cells, ROCK1/AMPK axis contributed to β-cell dysfunction under lipotoxic stress (127). Moreover, systemic ROCK inhibition increased whole body energy consumption and ameliorated metabolic disorders through AMPK activation in the skeletal muscle and liver (47, 48). Overall, numerous studies have suggested a detrimental role of ROCK1/AMPK signaling in nutrient metabolism in liver, skeletal muscle and pancreatic β-cells.

Different from ROCK1/AMPK axis mentioned above, AMPK has been reported as an upstream positive or negative regulator of RhoA/ROCK pathway through various context dependent mechanisms in smooth muscle cells (76, 128, 129), endothelial cells (130) and podocytes (131). For instance, AMPK activation by metabolic stress reduced smooth muscle contractility attributed to phosphorylating RhoA and inhibiting the RhoA/ROCK/MYPT1/MLC2 pathway (76). In contrast, Toll-like receptor 9-dependent AMPK activation contributed to RhoA/ROCK activation and actin polymerization in smooth muscle cells (128). It is worth noting that whether ROCK1/AMPK or AMPK/RhoA/ROCK/MYPT1/MLC2 pathways could play a significant role in metabolic functions of adipose tissues has not yet been documented.

### ROCK Isoforms Differentially Function in Various Cell Types

Though the two ROCK isoforms are very similar and are possibly somewhat redundant, a solid body of evidence indicates that they also have some unique functions. For instance, although both ROCK1 and ROCK2 control assembly of the actin cytoskeleton and cell contractility *via* phosphorylation of MYPT1, the mechanism may vary between the two. Only ROCK2 binds directly to and phosphorylates MYPT1 (132), suggesting that intermediate proteins are involved in ROCK1 binding to MYPT1. Moreover, these two ROCK isoforms differ in their binding capacities for IRS1 and only ROCK2 was found to bind to IRS1 although both isoforms are involved in insulin-stimulated phosphorylation of IRS1 at serine 632/635 (40). The *in vivo* functional similarity and differences of ROCK1 and ROCK2 have been shown in mouse genetic studies during embryonic development and under pathological conditions (133). For instance, genetic approaches have revealed pleiotropic actions of ROCKs in regulating insulin signaling and obesity, and the ultimate phenotype depends on ROCK isoforms and metabolic organs (30, 38, 41, 42, 121, 125, 134). Their functional differences could be explained by the facts that both isoforms are expressed at different levels and/or they have different interacting partners in individual cell types.

### Recent Advances in Metabolic Study of ROCK2 Isoform-Specific Inhibitors

The high sequence identity between ROCK1 and ROCK2, particularly in their kinase domain, makes the design of isoform­selective inhibitors very challenging (55, 56, 135). KD025 is the first highly selective ROCK2 inhibitor, and it achieved a good isoform selectivity of >200-fold for ROCK2 *versus* ROCK1 (60). Its therapeutic potential has been explored in fibrotic disease (60), focal cerebral ischemia (61, 136, 137), brain injury related to blood-brain barrier disruption (137), pulmonary arterial hypertension (138), and auto-immune disease (62, 139–144). Notably, the hypotensive phenotype was not observed when KD025 was tested in systemic application (61), therefore, emerging as an important breakthrough in systemic application. As a potential new drug candidate, KD025 is currently undergoing several phase 2 clinical trials for psoriasis, idiopathic pulmonary fibrosis, chronic graft-versus-host disease, and systemic sclerosis (142, 145, 146).

Recent studies explored therapeutic potential of KD025 in metabolic disease including renal fibrosis in the unilateral ureteral obstruction mice (147), arteriosclerosis and vascular fibrosis in a diabetic mouse model (148). Their therapeutic effects are partly due to the inhibition of profibrotic pathways and fat metabolism in both ROCK2-dependent and independent manners (149). Moreover, KD025 has been shown to inhibit adipogenesis in human adipose-derived stem cells, 3T3-L1 preadipocytes and human orbital fibroblasts through a ROCK2-independent mechanism (150–152), by inhibiting casein kinase 2 (153). In stromal vascular (SV) cells isolated from mouse subcutaneous WAT, we observed both pro-beige adipogenic (through ROCK2 inhibition) and anti-adipogenic (targets not clear) actions of KD025, which can be dissociated by dose- and time-dependent analyses during the *in vitro* differentiation process (154). A recent bioinformatics analysis confirmed the function of KD025 in regulating inflammation and adipogenesis pathways and revealed several novel regulatory functions, including oxidative phosphorylation, Wnt signaling, angiogenesis, and KRAS signaling (155). These studies revealed the presence of on-target (ROCK2-dependent) and off-target (ROCK2-independent) effects of KD025.

Owing to the off-target effects such as anti-adipogenic activity through inhibiting casein kinase 2 (153), KD025 may produce combinatorial effects on adipogenesis resulting from the dual inhibition of ROCK2 (promoting adipocyte differentiation) and CK2 (inhibiting adipocyte differentiation). Although the effects of KD025 on adipogenesis has been extensively studied in cell culture models, its therapeutic potential in reducing adiposity and improving insulin sensitivity has not yet been evaluated in experimental animals such as diet-induced obese mice or genetically obese mice. Furthermore, in a phase 2 study of patients with psoriasis vulgaris who were mostly obese with BMI > 30, giving oral administration of three different doses of KD025 for 3 months, didn’t see significant differences in the body weight among the dose-different groups (142), suggesting that specific studies focusing on obese and diabetic patients should be designed in order to rigorously evaluate its anti-obesity potential. Therefore, both experimental and clinical studies are required to evaluate the prospect of KD025 in prevention and treatment of obesity.

## Cellular Actions of ROCK Signaling Pathways in Adipose Tissue

Healthy white adipocytes maintain a remarkably high degree of plasticity, quickly responding to whole body energy demands either through the release of fatty acids and glycerol, storage of excess calories as triglycerides and secretion of adipokines. Unhealthy remodeling of adipose tissue, especially visceral WAT from overfeeding leads to adipocyte dysfunction, reduced adipogenic capacity, and tissue fibrosis, all of which can contribute to whole body metabolic dysfunction, including type 2 diabetes and cardiovascular complications. BAT has significant effects on whole-body energy homeostasis. Promoting BAT function and the induction of beige adipocyte formation in WAT, which is a hallmark feature of adipose tissue browning, have emerged as promising therapeutic targets to increase energy expenditure and counteract unhealthy remodeling of adipose tissue. In response to certain environmental, genetic or pharmacological stimuli, the newly formed beige adipocytes in WAT are derived from 1) the recruitment of beige adipocyte progenitors from perivascular progenitors, 2) differentiation of preadipocytes, and 3) the transdifferentiation from mature white adipocytes (**Figure 2**). It is worthy to note that recent lineage-tracing studies have revealed that instead of the transdifferentiation from mature white adipocytes, beige adipocytes can arise from reactivation of dormant beige adipocytes which acquire white adipocyte-like unilocular morphology after warm adaptation (156). Here, we focus on the current status of RhoA/ROCK research in regulating the recruitment of adipocyte progenitors and adipocyte differentiation for all three types of adipocytes, WAT browning, and WAT remodeling due to overfeeding.

### Lineage Commitment and Recruitment of Adipocyte Progenitors

Early studies support the contribution of RhoA/ROCK pathway to regulating a switch between adipogenesis and osteogenesis of mesenchymal stem cells (36) (**Figure 2**). Many local mechanical stresses and environment factors influence stem cells in developmental and adult contexts, including changes in cell shape, cytoskeletal tension and RhoA/ROCK signaling which are essential to the commitment of stem cell fate (35, 36, 157–159). Inhibition of RhoA/ROCK activity enhances adipogenic differentiation of human and murine bone marrow mesenchymal stem cells (36, 157, 158) and of human adipose tissue-derived stem cells (160). More recent studies support the contribution of MRTF-A/SRF to acting at downstream of the RhoA/ROCK pathway to promote murine bone marrow mesenchymal stem cell commitment (113) and human adipose tissue-derived stem cell commitment (161) to osteogenic versus adipogenic lineages. As mentioned above, by controlling expression of adipogenesis-related genes as well as cytoskeleton, focal adhesion, and extracellular matrix genes, MRTF-A/SRF inhibits adipogenesis. In addition, RhoA/ROCK-mediated actin cytoskeleton formation also promotes nuclear translocation of transcription factors yes-associated protein 1 (YAP) and transcriptional coactivator with PDZ-binding motif (TAZ), which promote osteogenesis and suppress adipogenesis (162). Moreover, a reciprocal enhancement of cell adhesion mediated by focal adhesion kinase activity and actin dynamics mediated by RhoA/ROCK signaling is involved in the commitment towards osteogenic versus adipogenic lineages of human adipose stem cells (163). In addition, RhoA/ROCK/AMPK/SREBP axis is involved in inhibiting adipogenesis and promoting osteogenesis during cell sheet formation of human adipose tissue-derived stem cells, which represents a cell culture model of spontaneous self-organization of multipotent mesenchymal stem/stromal cells (164). These studies support a negative role of RhoA/ROCK/F-actin/MRTF-A/SRF, RhoA/ROCK/F-actin/YAP/TAZ, or RhoA/ROCK/AMPK/SREBP pathway in the commitment of mesenchymal stem cells to the adipogenic lineage.

RhoA/ROCK pathway has also been shown to control a switch between adipogenesis and myogenesis of common mesenchymal precursors *in vivo* and *in vitro* (35) (**Figure 2**). In embryonic heart, ROCK inhibition could lead to fibrofatty replacement of cardiomyocytes in the right ventricle of adult mice resulting in arrhythmogenic right ventricular cardiomyopathy (165). Mechanistically, both Wnt/β-catenin and RhoA/ROCK pathways must be inactive for a significant increase of PPARγ expression, which is responsible for mediating arrhythmogenic right ventricular cardiomyopathy pathogenesis (166). Moreover, in embryonic heart, the RhoA/ROCK/F-actin/MRTF-A/SRF pathway is essential to maintaining cell-cell contacts of developing cardiomyocytes and inhibiting this pathway during cardiomyocyte differentiation primes cardiac progenitors to switch to the brown/beige adipocyte lineage in response to adipogenesis-inducing signals (114). Mechanistically, inhibition of RhoA/ROCK/F-actin/MRTF-A/SRF pathway due to impaired cell-cell contacts at the intercalated disc represses the MRTF-A/SRF‐regulated myogenic lineage commitment while increasing PPARγ gene expression and activation of beige adipogenic program. These studies support a negative role of the RhoA/ROCK/F-actin/MRTF-A/SRF pathway in directing the commitment of skeletal muscle progenitors and cardiomyocyte progenitors to the brown/beige adipocyte lineage.

In agreement with the contribution of RhoA/ROCK/F-actin/MRTF-A/SRF cascade in modulating recruitment of white as well as beige and brown adipocyte progenitors, a bone morphogenetic protein-7 (BMP7)-induced inhibition of RhoA/ROCK/F-actin/MRTF-A/SRF axis directs the commitment of adipose stromal progenitors to beige adipocyte over vascular lineages (16); knocking down SRF or MRTF-A/B in multipotent mesenchymal stem cells enhanced lineage commitment of mesenchymal progenitors to brown adipocyte lineage (115). Mechanistically, inhibition of SRF or MRTF-A/B attenuated TGF-β signaling pathway and increased BMP signaling pathway, suggesting a potential regulatory feedforward loop between MRTF/SRF and TGF-β/BMP for lineage specification in thermogenic adipocyte progenitors.

### Adipocyte Differentiation

Inhibition of RhoA/ROCK pathway not only promotes recruitment of adipocyte progenitors, but also facilitates preadipocyte differentiation into mature adipocytes (34, 37, 152, 167–170) (**Figure 2**). Similar downstream pathways and crosstalk pathways with RhoA/ROCK are involved in both lineage commitment and differentiation of adipocytes. For instance, a cross-talk between the Rho/ROCK pathway and the Wnt/β-catenin pathway controls differentiation of preadipocytes (168); MRTF/SRF signaling suppresses preadipocyte differentiation (37, 167). It should be mentioned that adipocyte differentiation is accompanied by a pronounced change in the actin cytoskeleton, which is characterized by the disruption of the F-actin stress fibers and their reorganization into cortical F-actin structures which are essential for the completion of adipocyte differentiation. Both the MRTF/SRF signaling pathway (negative regulator) and the insulin/PI3K/Rac1 pathway (positive regulator) control the formation of adipocyte-associated cortical actin structures (170).

There is also mounting evidence supporting a negative impact of ROCK activity on brown and beige adipocyte differentiation by multiple mechanisms. Early studies support the contribution of RhoA/ROCK/IRS pathway to the suppression of brown adipogenesis through inhibiting insulin signaling (171–173). More recent studies support the concept that the RhoA/ROCK/F-actin/MRTF/SRF signaling pathway inhibits brown and beige adipogenesis and PPARγ expression (16, 115, 154). Knocking down SRF or MRTF-A/B in brown preadipocytes enhanced brown preadipocyte terminal differentiation associated with attenuated TGF-β signaling pathway and increased BMP signaling pathway (115).

During adipogenesis of white, beige and brown adipocytes, the protein expression and activity of RhoA, ROCK, MRTF-A/B and SRF are down-regulated (16, 115, 154). Both cyclic AMP and cyclic guanosine monophosphate contribute to the downregulation of RhoA/ROCK signaling (171–173). Moreover, among other upstream regulators, the regulator of G protein signaling 2 suppresses Gq/RhoA/ROCK signaling and promotes brown adipogenesis (174). Furthermore, PPARγ increases Rho GTPase-activating protein (GAP) DLC1 expression leading to suppression of RhoA/ROCK signaling, this provides a regulatory feedback loop between PPARγ and RhoA/ROCK pathways during white and brown adipocyte differentiation (175).

As mentioned above, the established general concept is that the inhibition of RhoA/ROCK-mediated actin cytoskeleton dynamics is required for the recruitment of progenitors and differentiation of white, brown and beige adipocytes. Recent studies support the concept that the grade of RhoA/ROCK inhibition can differently regulate these three types of adipocyte differentiation. For instance, there is an inverse relationship between RhoA/ROCK activity and the abundance of brown/beige adipocytes in fat depots (154). Specifically, ROCK1 and ROCK2 protein expression, total ROCK activity, especially ROCK2 activity were the lowermost in BAT, in addition, they were lower in subcutaneous WAT compared to epididymal WAT (154). In addition, β-adrenergic stimulation, which enhances beige adipocyte formation in subcutaneous WAT, down-regulates RhoA/ROCK protein expression and activity, including ROCK2 protein expression and total ROCK activity without affecting ROCK1 protein expression (154).

A negative role for ROCK2 in regulating beige adipocyte formation has been supported by the observations that reduction in ROCK2 signaling poises differentiating adipocytes towards beige adipocytes *in vivo* and *in vitro* (154). Indeed, acquired data from either systemic *ROCK2^+/-^* mouse model, or from *ROCK2^+/KD^* mouse model in which mouse harboring an allele with a kinase-dead (KD) mutation, showed a lean body mass phenotype during aging and was associated with increased amounts of beige cells in subcutaneous WAT and increased thermogenic gene expression, including UCP1 in WAT and BAT (154). *ROCK2^+/-^* mice also exhibited increased sensitivity to the browning effects of β-adrenergic stimulation resulting in increased beige cell formation in subcutaneous WAT, and increased BAT mass. *In vitro* differentiated *ROCK2^+/-^* SV cells isolated from subcutaneous WAT or BAT exhibited increased beige or brown adipogenesis, respectively (154). It remains to be determined through cell type-specific knockout approach if ROCK2 activity inhibits the recruitment of beige adipocyte progenitors, differentiation of preadipocytes to beige adipocytes, and the transdifferentiation from mature white adipocytes *in vivo*.

### Adipose RhoA/ROCK Activation Contributes to Adipocyte Hypertrophy, Inflammation and Fibrosis

WAT undergoes expansion and extensive remodeling during diet-induced obesity, including increased adipose cell size (hypertrophy) and cell number (hyperplasia) after short-term overfeeding. This process is accompanied by the development of a chronic low-grade inflammation in adipose tissue, presenting as infiltration of immune cells and increased levels of pro-inflammatory cytokines, then followed by the development of tissue fibrosis after chronic overfeeding. Hypertrophic adipocytes are less responsive to insulin, and adipocyte size serves as a predictor for development of type 2 diabetes.

Mouse on HFD is linked with augmented RhoA/ROCK activity in adipose tissue where increased F-actin assembly and changed expression of actin-regulating proteins favor actin polymerization (29, 30, 154, 176), which further enhances adipocyte stretch due to excess lipid accumulation and creates a vicious cycle of adipocyte hypertrophy and dysfunction (29). Although there is a unanimous agreement on the increased total ROCK activity in adipose tissue from HFD-fed mice (29, 176), there are also discrepancies on increased isoform-specific activity (30, 121). For instance, in one study, a significant increase in ROCK2 activity without changes in its protein expression was reported in adipose tissue from HFD-fed mice, while ROCK1 protein expression and activity remained unchanged (121). But another study observed increased activity of both isoforms, ROCK1 and ROCK2 in adipose tissue of HFD-fed mice, however, no change was found in protein expression of either isoform (30).

Transgenic mice that overexpress an adipocyte-specific, dominant-negative form of RhoA showed decreased ROCK activity in adipocytes, decreased adipocyte hypertrophy and dysfunction, and reduced macrophages in adipose tissue (29). Adipocyte-specific ROCK1 knockout mice showed modest amelioration in insulin sensitivity and insulin signaling, but no significant effects on adipocyte hypertrophy and inflammation under HFD (30), suggesting that the ROCK2 isoform in adipose tissue may play a dominant role in controlling adiposity and insulin sensitivity. Indeed, partial deletion of ROCK2 in mice demonstrated diminished adipocyte hypertrophy, inflammation and fibrosis under HFD (121, 154). Future animal study with adipocyte specific ROCK2 knockout should help determine adipocyte autonomous function of ROCK2 *in vivo*. Mechanistically, downstream of RhoA/ROCK-mediated F-actin assembly, nuclear translocation of MRTF-A suppresses the expression of IRS1 and PPARγ in hypertrophic adipocytes, contributing to adipocyte dysfunction and insulin resistance (116). Moreover, RhoA/ROCK-mediated actomyosin contractility promotes nuclear translocation of the transcriptional co-activators YAP/TAZ which suppress expression of pro-apoptotic factor Bim and protect against white adipocyte cell death during obesity (177).

In addition to increasing adipocyte size, visceral WAT adipocytes respond to chronic overfeeding by adopting a fibroblast-like phenotype, which is characterized by enhanced expression of extracellular matrix proteins, focal adhesion and cytoskeletal genes, and suppression of many adipocyte programs most notably those associated with mitochondria (118). In obesity, visceral WAT adipocytes progressively become metabolically flawed due to the acquirement of fibrogenic functions. During this process, RhoA/ROCK/MRTF-A/SRF signaling within the adipocytes contributes to both upregulation of morphological genes as well as suppression of mitochondrial programs (118). Moreover, outside of adipocytes, RhoA/ROCK/MRTF-A/SRF activation within visceral WAT shifts the fate of perivascular progenitors to fibrogenic *versus* adipogenic progenitors (117). Therefore, both cellular mechanisms of RhoA/ROCK/MRTF-A/SRF signaling contribute to chronic obesity-induced fibrosis and metabolic dysfunction of adipose tissue.

### Adipose RhoA/ROCK Activation Contributes to Obesity and Insulin Resistance, and Inhibits Energy Expenditure

As mentioned above, transgenic mice overexpressing an adipocyte-specific, dominant-negative form of RhoA exhibited decreased visceral adipose tissue remodeling associated with decreased HFD-induced weight gain and improved glucose metabolism (29). Partial deletion of ROCK2 in mice also decreased adipose tissue remodeling associated with decreased HFD-induced adipose and systemic insulin resistance (121, 154). However, there is still discrepancy on the effects of partial deletion of ROCK2 on HFD-induced weight gain from studies using mice of different genetic background (42, 121, 154).

In addition to visceral WAT remodeling, we recently revealed a role for ROCK2 as a negative regulator of thermogenic programs in WAT and BAT through facilitating actin cytoskeleton assembly (154). *ROCK2* ^+/-^ mice on HFD demonstrated increased sensitivity to the browning effects of beta-adrenergic stimulation, increased energy expenditure with reduced obesity, and improved insulin sensitivity. In contrast to *ROCK2* ^+/-^ and *ROCK2* ^+/KD^ mice, *ROCK1* ^+/-^ and *ROCK1* ^+/KD^ mouse models didn’t exhibit a lean body mass phenotype (154) which is consistent with adipocyte-specific ROCK1 knockout mice with modest amelioration in systemic insulin sensitivity and adipose insulin signaling, but with no significant effects on body weight gain under HFD (30). Thus, inhibition of RhoA/ROCK2 in adipose tissue may provide a potential therapeutic strategy to combat obesity and insulin resistance (**Figure 1**).

## Metabolic Roles of Liver, Skeletal Muscle, Brain and Vascular RhoA/ROCK Signaling Pathways in Energy Expenditure, Diet-Induced Obesity and Insulin Resistance

Aforementioned both ROCK1 and ROCK2 are ubiquitously expressed across tissues in human and mouse (27). Importantly, up-regulated ROCK activity has been implicated in the pathogenesis of all aspects of metabolic syndrome including obesity, insulin resistance, dyslipidemia and hypertension (29–33). Here, we focus on the current status of RhoA/ROCK research in major metabolic organs that play vital roles in the development of obesity and insulin resistance. We also devoted special attention to the impacts of RhoA/ROCK activation in liver, skeletal muscle and brain on adipose tissue remodeling and energy expenditure through paracrine mechanisms (**Figure 1**).

### Metabolic Roles of Liver RhoA/ROCK Signaling

Both liver and skeletal muscle are major metabolic tissues involved in the regulation of whole-body glucose, lipid and energy homeostasis. Systemic overexpression of dominant-negative ROCK in mice or systemic ROCK inhibition with fasudil improved HFD-induced metabolic disorders including obesity, hypercholesterolemia and glucose intolerance in mice (47, 48). These metabolic improvements were mediated through the activation of AMPK pathway in liver and skeletal muscle which contributed to increased whole body energy expenditure (47, 48). In addition to systemic metabolic improvements, treatment with ROCK inhibitors ameliorates liver fibrosis in diabetic non-alcoholic steatohepatitis (178–180).

It is noteworthy that different from adipose tissues where ROCK2 appears to the major isoform contributing to ROCK activity, ROCK1 is reportedly the major isoform in liver and mediating diet-induced obesity and insulin resistance through the ROCK1/AMPK signaling pathway (47, 48, 59, 125, 181) (**Figure 1**). Liver-specific knockout of ROCK1 mice were resistant to diet-induced obesity due to increased energy expenditure and thermogenic gene expression in adipose tissue, whereas hepatic overexpression of ROCK1 was sufficient to promote adiposity, insulin resistance, and hepatic lipid accumulation in mice fed with a HFD (125). In addition, ROCK1/AMPK/SREBP1c axis was reported to regulate hepatic lipogenesis and contribute to nonalcoholic fatty liver diseases (125). Furthermore, a paracrine mechanism through liver-released hepatokines including FGF21, increased in liver of hepatic ROCK1 knockout mice, potentially mediating the effects of hepatic ROCK1 knockout on inducing thermogenic gene expression in adipose tissue and increasing energy expenditure (125). Although these studies established a role for hepatic ROCK1 in promoting adiposity, insulin resistance, and hepatic lipid accumulation, the role for hepatic ROCK2 in regulating these metabolic functions remains to be explored.

### Metabolic Roles of Skeletal Muscle RhoA/ROCK Signaling

In skeletal muscle, ROCK1 and total ROCK activity have been reported to play either positive or negative metabolic roles depending on metabolic conditions (38, 41, 126, 182–185) (**Figure 1**). On one hand, ROCK-mediated IRS1 phosphorylation in skeletal muscle can positively impact insulin signaling under normal diet or in exercised animals (38, 41, 183, 184). On the other hand, ROCK1/AMPK/SREBP1c and ROCK/IRS1 pathways have been found involved in lipotoxicity and insulin resistance on HFD (126, 185). A finding indicated that increased ROCK1 activity in skeletal muscle through muscle-specific expression of constitutive ROCK1 reduced irisin expression in muscle resulting in a low level of irisin in circulation. Low level of irisin suppressed UCP1 expression in BAT and subcutaneous WAT through a paracrine mechanism (134). Irisin administration to these mice partially reversed insulin resistance and obesity, and these changes were associated with increased expression of UCP1 in subcutaneous WAT (134). Hence, the reduced adipocyte browning contributes to reduced heat production, development of obesity and insulin resistance in transgenic mice. It remains to be determined if ROCK2 activation in skeletal muscle also regulate these metabolic functions.

### Metabolic Roles of Central Nervous System RhoA/ROCK Signaling

Different from liver where ROCK1 activation plays detrimental metabolic roles, ROCK1 plays a positive role in leptin signaling in the brain which controls feeding behavior, energy expenditure, and glucose metabolism (58, 186, 187) (**Figure 1**). Diet-induced and genetic forms (db/db and ob/ob) of obesity were associated with reduced ROCK1 activity in murine hypothalamic neurons (187). In addition, mice lacking ROCK1 in pro-opiomelanocortin and agouti-related protein neurons, which are mediators of leptin action, displayed obesity and impaired leptin sensitivity associated with increased food intake, reduced energy expenditure and locomotor activity (186, 187). Mechanistically, the leptin/ROCK1/JAK2 pathway promotes downstream signaling pathways of leptin including Stat3 and PI3K signaling in hypothalamic neurons (186, 187). Moreover, mice lacking RhoA in hypothalamic tyrosine hydroxylase neurons showed increased sensitivity to ghrelin and decreased sensitivity to leptin, resulting in increased food intake and development of obesity (188). It must be noted that systemic *ROCK1* ^-/-^, *ROCK1* ^+/-^ and *ROCK1* ^+/KD^ mouse models did not reveal overeating behavior and increasingly gained body weight compared to control mice (38, 154), suggesting that the central effects of ROCK1 deficiency in hypothalamic neurons are compensated by opposite metabolic effects of ROCK1 deficiency in other cells and organs. It remains to be determined through cell type-specific knockout approach as performed for ROCK1 if ROCK2 in central nervous system regulates food intake and energy expenditure.

### Metabolic Roles of Vascular RhoA/ROCK Signaling and Metabolic Effects Of Systemic Inhibition of RhoA/ROCK Signaling

Fasudil is a non-isoform selective inhibitor approved for human use in Japan for cerebral vasospasm after surgery of subarachnoid hemorrhage (189, 190). Studies using ROCK inhibitors in animal models of obesity, diabetes, and associated complications have demonstrated beneficial outcomes (29, 43–53). However, ROCK inhibitors have not yet been accepted for treating metabolic disorders due to potential systemic side effects, including hypotension caused by smooth muscle relaxation due to both ROCK1 and ROCK2 are similarly inhibited (54–56, 191).

In recent studies described above, the major organs mediating the beneficial metabolic effects of systemic ROCK inhibition includes adipose tissues (29, 121, 154), liver (47, 48, 178, 180), skeletal muscle (47, 48), hearts (42, 192) and intestine (193). In earlier studies, the beneficial effects have been focused on the vascular systems. For instance, ROCK inhibitor treatment reduced atherosclerotic lesion formation and vascular dysfunction in diabetic mice (49, 194–196). Mechanistically, perivascular adipose tissue in HFD obese mice releases reactive oxygen species (ROS), vasoconstrictors and proinflammatory factors, which in turn increase RhoA/ROCK-mediated vascular smooth muscle cell (VSMC) contractility and vascular remodeling (197–199). In addition, hyperglycemia, hyperlipidemia and advanced glycation endproducts activate RhoA/ROCK pathway in endothelial cells and VSMCs. This results in endothelial dysfunction and increased contractility, proliferation, migration and production of extracellular matrix proteins of VSMCs leading to obesity-induced hypertension and atherosclerosis (148, 200–204).

## Growing Research Areas in RhoA/ROCK Signaling With Metabolic Implications

In addition to the established research areas described above, there are several rapid growing research areas on RhoA/ROCK signaling with noticeable implications on metabolic regulation. Here, we focus on the current understanding of RhoA/ROCK research on circadian rhythm, mitochondrial function, mitophagy, and miRNAs. Future studies are expected to fully explore their regulation and function in metabolic tissues.

### Metabolic Association of RhoA/ROCK Signaling With Circadian Rhythm

Circadian rhythm exhibits 24-h cycles in the body, and metabolic processes are under circadian regulation. Disruption of the circadian rhythms can disrupt clock gene expressions in both central (e.g., suprachiasmatic nucleus) and peripheral tissues (e.g., adipose tissue, liver and vasculature) causing dysfunction in energy metabolism and nutrient metabolic homeostasis, and leading to development of metabolic disorders including obesity, diabetes, dyslipidemia and hypertension (205). Numerous studies support a role for ROCKs in regulating circadian rhythm of vascular contractility, giving rise to blood pressure circadian rhythm, which is essential for cardiovascular health (206, 207). For instance, the circadian rhythm of ROCK1 and ROCK2 expression in vascular smooth muscle was disrupted in db/db obese mice (208). In addition, ROCK2 played a pivotal role in generating the intrinsic circadian rhythm of vascular contractility in the mouse aorta as ROCK2 expression exhibited the circadian oscillation in phase with that of MLC2 phosphorylation (209, 210). Moreover, an altered circadian ROCK activity in circulating leukocytes was associated with alterations in coronary vasomotor responses and autonomic activity in vasospastic angina patients (211). Mechanistically, the circadian ROCK2 expression in vasculature is under the control of clock genes RORα and Bmal1 as both activate the transcription of ROCK2 (209, 212). ROCK1 expression was found negatively regulated by clock gene CLOCK in human umbilical vein endothelial cells (213). In addition to being transcriptionally regulated by clock genes, RhoA/ROCK signaling can act upstream and regulate clock gene activity. For instance, RhoA/ROCK-mediated mechano-sensing to extracellular stiffness regulates the activity of the core circadian clock complex in primary breast epithelial cells (214). The role RhoA/ROCK signaling in controlling or mediating circadian rhythm in other metabolic tissues currently remains to be explored.

### Impacts of RhoA/ROCK Signaling on Mitochondrial Function and Mitophagy

The mitochondria play critical roles in oxidative phosphorylation and energy metabolism in all metabolic tissues. As described above, RhoA/ROCK signaling contributes to the regulation of thermogenic gene expression and mitochondrial biogenesis in adipose tissue. Inhibition of RhoA/ROCK signaling results in increased PPARγ expression, which promotes thermogenic gene expressions and energy expenditure in adipose tissue (**Figure 1**). In liver and skeletal muscle, inhibition of RhoA/ROCK signaling induces thermogenic gene expression in adipose tissue through a paracrine mechanism (**Figure 2**). Due to the critical roles of mitochondria in regulating cellular and systemic energy homeostasis, it is necessary to discuss some recent advances in our understanding on the impacts of RhoA/ROCK signaling to mitochondrial biology.

RhoA/ROCK signaling has been reported to function both upstream and downstream of mitochondria. On one hand, the mitochondria/ROS/RhoA/ROCK pathway is well-documented, which is trigged by mitochondrial dysfunction leading to the production of excessive ROS and subsequently RhoA/ROCK activation. For example, impaired mitochondrial function in the perivascular adipocytes of obese mice produced excessive ROS which triggered RhoA/ROCK activation resulting in increased vascular contractility (198, 215). Similarly, a critical mechanism contributing to the development of pulmonary hypertension in vascular hypercontraction is hypoxia. Under this condition, excessive ROS was produced by affected mitochondria that triggered activation of RhoA/ROCK signaling (216, 217). On the other hand, the RhoA/ROCK/dynamin-related protein-1 (Drp1) and F-actin/mitochondrial fission/ROS/apoptosis pathway has been reported in various diseases including diabetes, hypertension and Parkinson’s disease. For example, hyperglycemia-induced mitochondrial fission and dysfunction in endothelial cells is mediated by activating the RhoA/ROCK1/Drp1 signaling pathway, thus contributing to vascular complications of diabetes (218–220). This pathway also contributes to hypercontraction of VSMCs involved in artery constriction (221), cardiac inflammatory injury (222, 223), and dopaminergic nerve cell apoptosis in Parkinson’s disease (224).

Interestingly, inhibition of RhoA/ROCK pathways can also improve mitochondrial quality control by activating mitophagy. In the context of Parkinson’s disease, recent evidence supports beneficial effects of ROCK inhibition in promoting the Parkin-mediated mitophagy pathway which removes damaged mitochondria in dopaminergic neurons (225, 226). Mechanistically, ROCK2 inhibition promotes the activity of this pathway by increasing the recruitment of hexokinase 2, a positive regulator of Parkin, to mitochondria. This recruitment enables increased docking of impaired mitochondria to lysosomes, subsequently enhancing the removal of damaged mitochondria from cells (225, 226). In addition to the neuronal ROCK2/Parkin/mitophagy axis, the ROCK1/Parkin/mitophagy pathway was reported to mediate cisplatin-related resistance in lung cancer cells (227). It will be interesting to further explore if the ROCK/Parkin/mitophagy axis plays a role in regulating mitochondrial quality control in metabolic diseases like obesity and diabetes in future studies.

### miRNAs Contribute to Isoform-Selective Activation and/or Inhibition of RhoA/ROCK Signaling in Metabolic Tissues

Aforesaid recent research has identified miRNAs functioning as endogenous physiologic activators and/or inhibitors of ROCK1 and ROCK2 to achieve isoform-selective activation or inhibition in metabolic tissues (**Table 1**). The upstream activators of ROCK are affected by changing miRNAs expression levels (**Table 1**). For instance, when miR-125a-3p expression was up-regulated which then targeted RhoA leading to a down-regulation of RhoA/ROCK activity and followed by increased adipogenesis in subcutaneous WAT in patients with multiple symmetric lipomatosis (79). On the other hand, when miR-133a expression in gastric smooth muscle of ob/ob mice was down-regulated, as the result of RhoA was targeted, there was an increase in RhoA/ROCK signaling and muscle contraction (78). Furthermore, down-regulation of miRNA-141 was observed in penile cavernous smooth muscle cells of rats with diabetic erectile dysfunction, and the decreased expression of miRNA-141 was also associated with up-regulation of RhoA/ROCK signaling (86). Even though miRNAs expression changes are frequently linked to up- or down-regulation changes in RhoA/ROCK signaling pathway, the metabolic outcome is detrimental in most cases.

Known that ROCK1 and ROCK2 have different miRNA-binding sites, therefore, each isoform is individually up- and down-regulated based on available data (**Table 1**). ROCK1 was found to be a target of miR-146a-5p which was down-regulated in liver tissue taken from models of a nonalcoholic fatty liver; miR-146a-5p down-regulation in these models promoted lipid accumulation in hepatocytes through inhibiting adenosine monophosphate-activated protein kinase (AMPK) pathway (80). Down-regulation of miR-145 was also observed in cultured human vascular smooth muscle cells (VSMCs) exposed to high glucose; miR-145 down-regulation promoted ROCK1 expression and increased proliferation and migration of VSMCs which are important steps in diabetic atherosclerosis (89). In circulating peripheral blood and liver tissue of db/db mice, raised miR-324-5p expression was associated with hyperglycemia or hyperlipidemia leading to impaired glucose and lipid metabolism due to suppressed ROCK1 expression (81). During period of physical inactivity, the increased skeletal muscle miR-148b expression triggered the downregulation of ROCK1 expression and insulin resistance in humans and mice (82). In contrast to the detrimental effects of miRNA expression fluctuations in the metabolic tissues, up-regulation of miR-217 was found to be beneficial in both VSMCs treated by high glucose and in aorta VSMCs of diabetic rats, miR-217 up-regulation suppressed ROCK1expression in these cases and inhibited excessive proliferation and migration of VSMCs (90).

ROCK2, instead, was found to be a target of miR-455-3p which was down-regulated in the human mesangial cells and human proximal tubule epithelial cells when stimulating with high glucose or transforming growth factor beta 1 (TGF-β1); restoring miR-455-3p expression in a rat diabetic nephropathy model decreased ROCK2 expression and suppressed renal fibrosis (83). Down-regulation of miR-10a and miR-139b in VSMCs induced ROCK2 expression and contributed to diabetes and hyperlipidemia-induced vascular hyperreactivity (77). Lastly, some miRNAs target both ROCK1 and ROCK2, such as miR-497, which was increased in glomerular endothelial cells of diabetic rats and contributed to endothelial-to-mesenchymal transition and albuminuria through suppressing ROCK1 and ROCK2 (91). Integrative analysis of gene expression profiles including miRNAs and their target gene network in metabolic tissues has contributed significantly to our understanding of insulin resistance and obesity (228). Future studies in this area are expected to further expand the list of miRNAs targeting RhoA/ROCK pathways in metabolic diseases (**Table 1**) and elucidate their biological role at mechanistic level.

## Concluding Remarks

Studies using chemical inhibitors, whole-body and tissue-specific genetic manipulations in animal models have demonstrated that abnormal RhoA/ROCK signaling contributes to the pathogenesis of obesity and type 2 diabetes. These studies have identified that the ROCK isoforms have critical functions in various metabolic tissues, including adipose tissue, liver, skeletal muscle, and hypothalamus where they regulate thermogenesis, glucose and lipid metabolism, food intake, and locomotor activity (**Figure 1**). Significant progress has been made in dissecting the molecular and cellular mechanisms underlying these aberrant global or tissue-specific metabolism in which ROCK inhibition mainly shows beneficial outcome. In adipose tissue, ROCK inhibition provides favorable effects through reducing WAT remodeling under metabolic stress conditions, promoting BAT thermogenic function and browning process of WAT, leading to increased energy expenditure and reduced adiposity. Metabolic beneficial effects of ROCK inhibition are consistently observed in liver, skeletal muscle, vascular system and kidney under metabolic stress conditions. In contrast, ROCK inhibition in skeletal muscle and hypothalamus is metabolically detrimental under basal condition. These tissue-specific beneficial and detrimental effects of ROCK inhibition highlight the importance of tissue-specific targeting of ROCK activity *via* innovative delivery strategies (229–232).

Considering recent advances in understanding RhoA/ROCK signaling in regulating metabolic functions, promoting brown adipogenesis and browning process of WAT by ROCK inhibition, particularly ROCK2 inhibition, have emerged as promising strategies to treat obesity and its related metabolic disorders. However, a ROCK2-selelective inhibitor KD025, initially bearing great therapeutic potential, has been discovered to exhibit an anti-adipogenic activity through inhibiting casein kinase 2, a ROCK2-independent mechanism. With this off-target effect, it makes KD025 possibly unsuitable as a molecular tool to enhance thermogenesis.

We anticipate that future research in the field has three main directions (1): continuous dissection of molecular mechanisms and cellular functions of RhoA/ROCK signaling in the pathogenesis of obesity and type 2 diabetes with increased insights into mitochondrial dysfunction and circadian rhythm disruption; (2) continuous development of ROCK isoform-selective inhibitors and exploration of the endogenous physiologic activators and inhibitors of ROCK1 and ROCK2 such as miRNAs to achieve isoform-selective activation or inhibition in metabolic tissues; and (3) increasing efforts on studying RhoA/ROCK pathways in human obesity and other related metabolic disease, which is pathophysiological more complex than rodent models. Together, all these research efforts will improve our understanding of RhoA/ROCK signaling in thermogenesis and energy expenditure with the translational goal of developing new treatment for obesity and related disease.

## Author Contributions

JS and LW contributed to the idea for the article, performed the literature search and data analysis, drafted and revised the article. JS and LW agreed with the content of this manuscript and have approved the manuscript before submission.

## Funding

This work was supported by National Institutes of Health grants HL151480 (to LW and JS), HL107537 (to LW), HL134599 (to LW), Riley Children’s Foundation (RCF) (to JS and to LW), the Indiana Clinical and Translational Sciences Institute (CTSI) including Biomedical Research Grant of Indiana University School of Medicine 2286128 (to JS) and the Pilot Funding for Research Use of Core Facilities 2286118 (to JS).

## Conflict of Interest

The authors declare that the research was conducted in the absence of any commercial or financial relationships that could be construed as a potential conflict of interest.

## Publisher’s Note

All claims expressed in this article are solely those of the authors and do not necessarily represent those of their affiliated organizations, or those of the publisher, the editors and the reviewers. Any product that may be evaluated in this article, or claim that may be made by its manufacturer, is not guaranteed or endorsed by the publisher.

## Abbreviations

Actin filaments, F-actin; AMPK, adenosine monophosphate -activated protein kinase; BAT, brown adipose tissue; BMP, bone morphogenetic protein; Drp1, dynamin-related protein-1; G-actin, globular actin; GAP, GTPase-activating protein; GEF, guanine nucleotide exchange factor; HFD, high-fat diet; IR, insulin receptor; IRS1, insulin receptor substrate-1; KD, kinase-dead; miRNA, microRNAs; LIMK, LIM kinases; MRTF, myocardin-related transcription factor; MLC, myosin light chain; MYPT1, myosin binding subunit of myosin light chain phosphatase-1; PI3K, phosphatidylinositol 3-kinase; PPARγ, peroxisome proliferator-activated receptor γ; ROCK, Rho-associated coiled-coil-containing protein kinase; ROS, reactive oxygen species; SRF, serum response factor; SREBP1c, sterol regulatory element-binding protein-1c; SV, stromal vascular; TGF-β1, transforming growth factor beta 1; TAZ, transcriptional coactivator with PDZ-binding motif; UCP1, uncoupling protein-1; VSMCs, vascular smooth muscle cells; WAT, white adipose tissue; YAP, yes-associated protein 1.
